# Predation affects body shape in the knife livebearer *Alfaro cultratus* (Cyprinodontiformes: Poeciliidae)

**DOI:** 10.1002/ece3.10787

**Published:** 2023-12-06

**Authors:** Diego A. Ardon, Kaitlyn B. Golden, Trevor J. Williams, Mark C. Belk, Jerald B. Johnson

**Affiliations:** ^1^ Department of Biology Evolutionary Ecology Laboratories Provo Utah USA; ^2^ BYU Life Science Museum Brigham Young University Provo Utah USA

**Keywords:** Costa Rica, geometric morphometrics, knife livebearer, mixed model analysis, predation

## Abstract

Livebearing fishes are a common model for studying the effects of predation on prey biology. Numerous studies have found differences in life history, sexual selection, behavior, and morphology between populations of the same species that co‐occur with predators and those that do not. *Alfaro cultratus* is a livebearing fish with populations in different predation environments, but unlike other livebearers, this species also has an extreme body shape that is laterally compressed. Given this unusual morphology, we asked if predation environment would still predict overall body shape, as has been documented in other species. We collected specimens from both predator and no predator sites in Costa Rica and used a geometric morphometrics analysis to determine if body shape is affected by predation environment, while controlling for size and river gradient. Body shape does indeed differ between predation environments; however, the observed differences contrast with the patterns found in other livebearer systems. *Alfaro cultratus* in predation environments had deeper and shorter bodies and deeper caudal peduncles than those found in environments without dominant fish predators.

## Graphical abstract

The effects of predation on the morphology of livebearer species have been widely documented for round‐bodied forms; however, little is known on how this selective pressure affects more extremely shaped species. The narrow‐body species *Alfaro cultratus* inhabits both high and low‐predation environments and morphological comparisons among populations show that there is an effect of predation in body shape for this species. However, the morphological differences detected contrast with the effects in other livebearer systems. We hypothesize that this unique morphological effect is a product of *A. cultratus* narrow body.

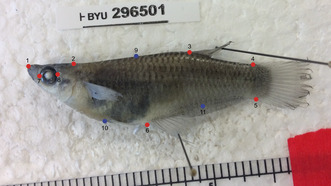

## 1 INTRODUCTION

Body shape in fish can be an important target for natural selection, resulting in a shape that represents the response to multiple selective pressures over time (Robinson & Wilson, 1994; Schluter, 1993; Smith & Skulason, 1996). For example, in fish, morphology can be related to predation environment (Blake, 2004; Brönmark & Miner, 1992; Webb, 1984); diet (Andersson et al., 2006; Jonsson & Jonsson, 2001; Merigoux & Ponton, 1998; Ruber & Adams, 2001; Smith & Skulason, 1996; Williams et al., 2017); swimming speed, agility, and stamina (Domenici, 2010; Helfman et al., 2009; Lauder & Drucker, 2002; Webb, 1984); competition (Schluter, 1993; Schluter & McPhail, 1992); and water velocity (Aguirre & Bell, 2012; Blake, 2004; Haas et al., 2015; Landy & Travis, 2015; Langerhans, 2008; Meyers & Belk, 2014; Zúñiga‐Vega et al., 2007).

Livebearing fishes (family Poeciliidae) have been widely studied to understand body shape variation, especially in response to predation environment (Hassell et al., 2012; Ingley et al., 2014; Johnson & Belk, 2020; Langerhans et al., 2004; Langerhans & DeWitt, 2004; Wesner et al., 2011). In general, fish from predator populations exhibit a larger caudal region, smaller head, more elongated body, and posterior‐ventral eye position relative to predator‐free populations (Langerhans et al., 2004). Later studies showed that these patterns hold in other species across the family (Ingley et al., 2014; Langerhans & DeWitt, 2004; Langerhans & Makowicz, 2009), suggesting a pattern of converging evolutionary divergence among populations and species.

However, more recent studies show that body shape is a complex trait that responds simultaneously to more than one selective pressure, often reflecting a trade‐off between optimal shapes for different pressures (Burns et al., 2009; Williams et al., 2017). In low predation systems, competition is the more important selective agent, but when a predator is present, avoiding predation becomes the most important pressure (Langerhans, 2009). In *Brachyrhaphis rhabdophora*, body shape differs between predator and non‐predator populations, but pregnant females tend to converge on a common shape, demonstrating a trade‐off between reproduction and optimal shape for survival (Wesner et al., 2011). These data suggest predation is important to the evolution of fish body shape, but most of the studies on variation in body shape as affected by predation environment are limited to a relatively small number of taxa with a typical round‐bodied (in cross section) form (Ingley et al., 2014; Langerhans & DeWitt, 2004; Langerhans & Makowicz, 2009). Unfortunately, we know almost nothing about fishes that have narrow‐bodied shape, which might have evolved as a response to selective pressures other than predation and may not fit the standard expected morphological predictions (Belk et al., 2011). What is needed are systems that allow us to examine effects of predation environment on body shape in fishes with more extreme body forms.

*Alfaro cultratus* presents a good system for evaluating the effect of predation on body shape in a poecilid with an atypical morphology. This species is highly laterally compressed with the lower margin of the caudal peduncle sharpened with scales forming a keel; thus, the common name knife livebearer (Bussing, 2002). Interestingly, both males and females have this body shape, and females maintain it during pregnancy (Wesner et al., 2011). Populations inhabiting the Atlantic versant of Costa Rica include systems with the presence of piscivorous predators including *Parachromis dovii*, and *P. managuensis* (high predation environment), and systems with few or no predators (low predation environment). Body shape response to predation across both types of environments might be analogous to that found in other livebearer systems (Ingley et al., 2014; Langerhans & DeWitt, 2004; Langerhans & Makowicz, 2009), it might show differences in body shape that are not analogous to other livebearer systems due to the interaction of predation with other selective pressures or there might be no differences in body shape between predation environments. For example, little variation in life history traits in *A. cultratus* was found between high and low predation environments (Golden et al., 2021), a contrast to the life history pattern found in other livebearer species (Johnson & Belk, 2001; Johnson & Zúñiga‐Vega, 2009; Reznick & Travis, 2019). This absence of divergence in life history between predation environments can be attributed to a limitation imposed by the compressed body shape of *A. cultratus*. This adaptation to a high velocity environment could in turn hinder the divergence in response to life history variations even in the presence of differing predation pressures among populations (Golden et al., 2021). A corollary implication is that body shape might remain consistent across predation environments due to the shared constraint from having a body adapted to high‐velocity environments.

Here, we tested whether body shape in *A. cultratus* diverges in response to predation environment and if that divergence is consistent with what has been reported for other livebearer species (Belk et al., 2020; Ingley et al., 2014; Langerhans et al., 2004; Langerhans & DeWitt, 2004) in spite of its atypical shape. Specifically, we test if, in the presence of predators, fish had a larger caudal region, smaller head, more elongate body, and posterior‐ventral eye position, relative to fish from predator‐free environments.

## 2 MATERIALS AND METHODS

### 2.1 Study site and collection

**FIGURE 1:**
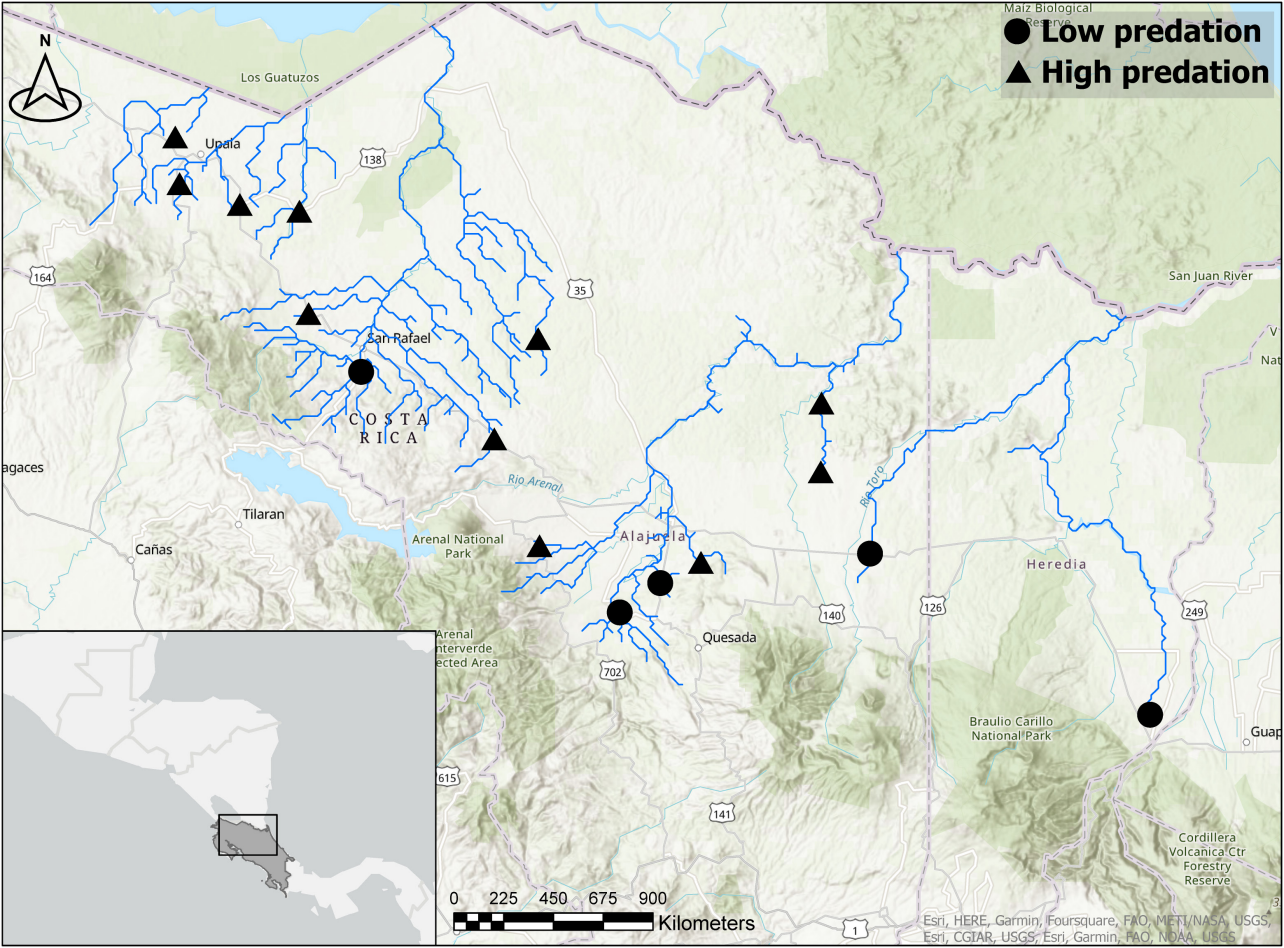
Collection locations in Costa Rica for high and low predation populations of *Alfaro cultratus*.

We collected *A. cultratus* individuals from 16 different sites in Costa Rica (see Figure 1 and Table 1) using a handheld seine (1.3 × 5 m; 8 mm mesh size), attempting to collect approximately 100 females for a life history study (Golden et al., 2021), a good proportion of which were adults used in the present study (see Table 1 for sample sizes for each sampling location). We categorized five of these locations as low predation environments (i.e., no piscivorous fishes were present) and 11 locations as high predation environments in a binary way, analogous to the classic Trinidadian guppy system (Reznick & Endler, 1982). We defined predation environments as “high predation environments” if we caught or observed either or both *P. dovii* or *Parachromis managuensis* (Bussing, 2002) during the sampling for *A. cultratus* specimens, and as “low predation environments” if we did not. Ten or more seine hauls were carried out at each location. We designated both site types as “high” or “low predation environments” but acknowledge that predation risk may be confounded with other environmental factors like resource availability, elevation, temperature, river flow, and density and that presence of predators may be causally or incidentally correlated to these or other factors (Johnson, 2002; Jourdan et al., 2016). Other forms of predation on fishes likely exist at these sites, including bird and invertebrate predation, but we did not account for their presence and densities in this study. Although some researchers have highlighted that predation should be studied as a gradient that considers temporal and spatial variation in predation risk (Deacon et al., 2018), classifying localities in a binary way has been shown to accurately predict mortality rates and divergent life history traits in the Costa Rican livebearer *B. rhabdophora* (Johnson, 2002; Johnson & Belk, 2001; Johnson & Zúñiga‐Vega, 2009), so although we did not measure mortality directly, we are using presence and absence of known fish predators as a predictor of mortality. We also calculated stream gradient at each location and used this factor as a covariate in our analyses (see below). The stream gradient was calculated using geographic information systems to calculate the difference in elevation (in m) over 1000 m stream length (500 m upstream and 500 m downstream of the collection site). The difference in elevation was divided by 1000 m and multiplied by 100 to obtain percent gradient. We consider gradient to be a predictor of river flow velocity, a factor associated with body shape in other fish species (Haas et al., 2015; Mercer, 2020). Low predation sites ranged in gradient from 2.29% to 7.75% and high predation sites from 3.02% to 6.14%.

**TABLE 1:** Locality information.

ID	Locality name	Geographical coordinates	Predation	Gradient	Sample size	Size range (mm)
19‐05	Rio Queque	10.64537, −84.822	Low	7.75	23	30.04–41.95
19‐06	Rio Guayabito	10.715, −84.8833	High	4.84	29	25.51–36.67
19‐07	Rio Zapote	10.866442, −85.033533	High	3.18	32	25.87–47.6
19‐08	Quebrada Las Latas	10.920701, −85.038569	High	5.13	38	22.98–43.2
19‐09	Rio Ricardo	10.842162, −84.963382	High	4.24	26	31.94–43.79
19‐10	Rio Rito	10.83433, −84.893977	High	4.95	24	27.66–37.71
19‐12	Rio Sabogal Tributary	10.685796, −84.61604	High	3.91	23	24.57–49.24
19‐13	Rio Pataste	10.569104, −84.4841833	High	6.14	36	28.75–47.74
19‐14	Quebrada Piedra	10.444626, −84.614434	High	4.54	29	23.91–44.59
19‐15	Rio Balsa Tributary	10.36519, −84.5209	Low	4.38	28	28.06–41.68
19‐16	Quebrada Serena	10.39939, −84.4738	Low	5.28	34	28.21–43.01
19‐17	Rio San Rafael Tributary	10.425327, −84.426494	High	4.88	25	26.75–41.42
19‐18	Quebrada Sahino	10.433683, −84.229512	Low	2.90	38	30.04–41.95
19‐19	Rio Sucio	10.24603, −83.9034	Low	2.29	14	28.15–42.61
19‐20	Quebrada Huevo	10.530829, −84.286938	High	4.15	37	27.72–41.45
19‐21	Rio Saino	10.610921, −84.286128	High	4.98	32	25.51–38.25

All fish were collected in April 2019 under Brigham Young University Institutional Animal Care and Use Committee approval (protocol #15‐0404). We conducted this work with permission from the Vida Silvestre, Sistema Nacional de Áreas de Conservación in Costa Rica (R‐SINAC‐PNI‐ACAHN‐011‐2019). We euthanized collected specimens in the field with an overdose of 3‐amenobenzoic acid ethyl ester (MS‐222) and then preserved them in 95% ethyl alcohol. Once transported to the laboratory, we stored specimens in 70% ethanol. We then measured and photographed each fish on the left side using an Apple iPad. We accessioned specimens into the Monte L. Bean Life Science Museum fish collections at Brigham Young University in Provo, Utah, USA.

### 2.2 Geometric morphometrics

**FIGURE 2:**
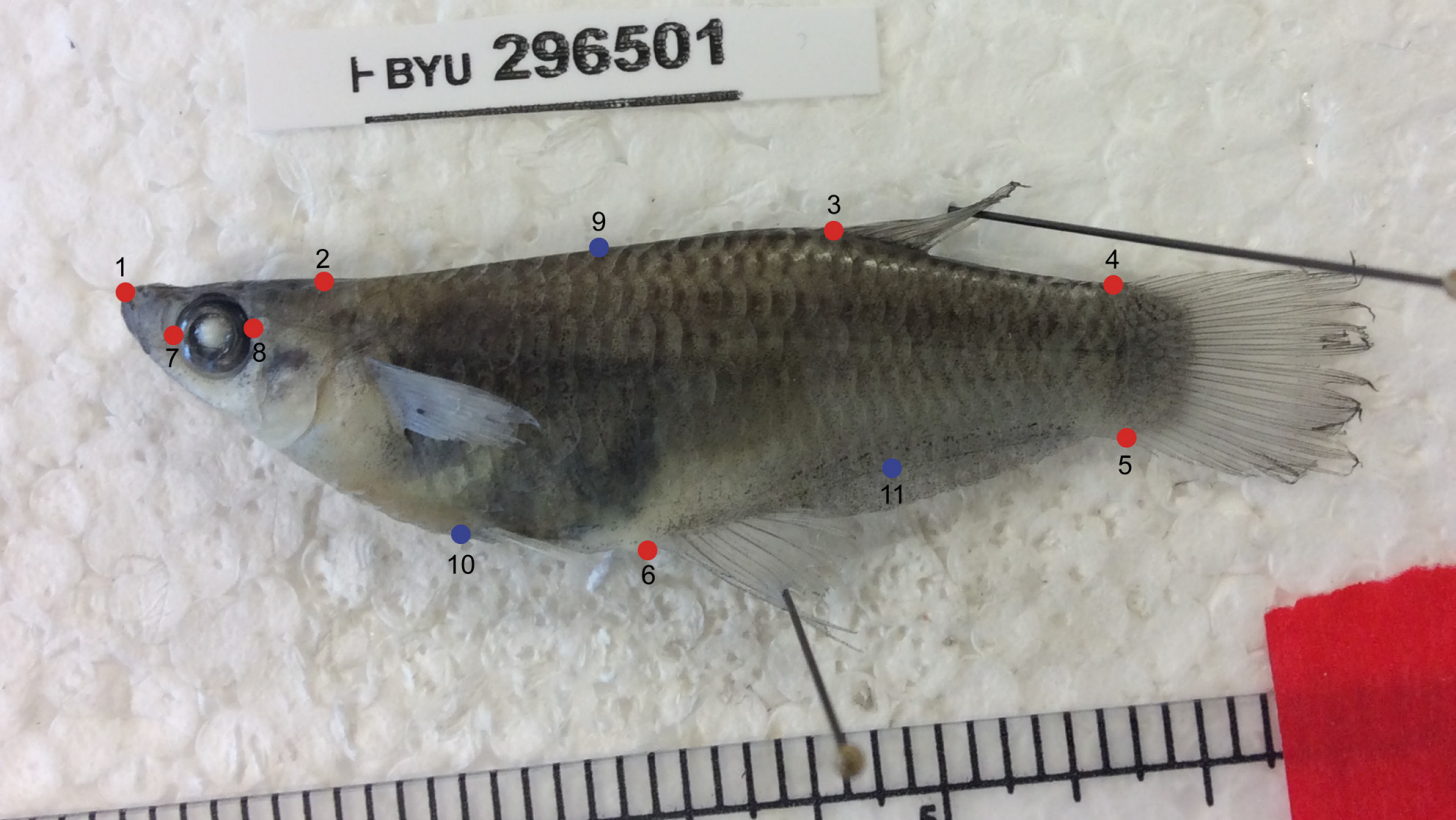
Photograph of *Alfaro cultratus* specimen with position of landmarks in red and semilandmarks in blue along the body.

We used 459 female specimens of *A. cultratus* for our analysis. Because *A. cultratus* is sexually dimorphic, we excluded males from the analysis. We photographed all specimens on their left side and digitized 11 landmarks to characterize body shape (Figure 2), using the software tpsDig (Rohlf, 2003a). Landmarks were (1) tip of the snout; (2) posterior extent of the operculum projected onto the dorsal outline; (3) anterior insertion of the dorsal fin; (4) dorsal insertion of the caudal fin; (5) ventral insertion of the caudal fin; (6) anterior insertion of the anal fin; (7) front of the eye; (8) back of the eye; (9) semilandmark on the dorsal outline halfway between landmarks 2 and 3; (10) semilandmark on the ventral outline at 2/3 the distance between landmarks 1 and 6; and (11) semilandmark on the ventral outline halfway between landmarks 5 and 6. A single person was responsible for digitizing all specimens, and digitizing was carried out without reference to the predictor variables (including the random effect of location). This procedure results in reduced digitization error and a random distribution of error compared to multiple individuals digitizing separate parts (i.e., locations or groups) of the data (Moccetti et al., 2023).

We used the software tpsRelW to align the specimens using a generalized Procrustes analysis to remove nonshape variation (Rohlf, 2015; Rohlf & Slice, 1990) and to generate shape variables for the images (Rohlf, 2003c). Shape variables were initially generated as partial warps and uniform components (**W** or weight matrix). The program then runs a principal component analysis of the weight matrix to generate relative warps to use as our measure for shape analysis. Relative warps are linear combinations of uniform and nonuniform shape components that are orthogonal to each other (Zelditch et al., 2012), and they capture multivariate shape variation in fewer dimensions. We used the first 12 relative warps (explaining 98% of shape variation) as response variables. This dimension reduction is important because of the use of sliding semi‐landmarks in the landmarking scheme. Landmarks carry two degrees of freedom, but sliding semi‐landmarks only have one true degree of freedom; thus, including all shape variables included in the original weight matrix creates far more degrees of freedom than are available in the data. Furthermore, we typically remove relative warps that individually account for <1% of shape variation. This reduction improves the likelihood of convergence for the parametric model (multivariate linear mixed model), and, again, guards against the inflation of degrees of freedom that individually account for none, or only a small amount, of the total shape variation.

### 2.3 Statistical analysis

We used a multivariate linear mixed model to determine the effects of predator environment on shape variation in *A. cultratus*. The response variable was shape as characterized by the first 12 relative warps. The predictor variables were predation environment, stream gradient (covariate), centroid size (covariate), and an index variable to account for the order of the relative warps and all two‐way interactions between predictors and the index variable. Size and gradient are known to affect body shape (Haas et al., 2015; Hassell et al., 2012; Langerhans, 2008; Meyer, 1990; Williams et al., 2017) in fishes, and although our samples exhibit little variation in both, we used centroid size (a multivariate measure of size) for each specimen and the gradient of the site as covariates. We specifically wanted to test for an effect of predation on body shape after adjusting for possible effects of body size (i.e., centroid size) and stream gradient (i.e., water velocity). Collection location was treated as a random effect, hence creating the need for a multivariate (multiple shape variables analyzed simultaneously) mixed (fixed and random effects) model.

A mixed‐model framework assumes a univariate response variable, so we vectorized the shape variables such that each row represented one response variable, but each specimen was represented by multiple rows of data (Anderson, 2003). Thus, the first row represented relative warp 1 for the first specimen, the second row represented relative warp 2 for the first specimen, and so forth until all relative warps were represented in successive rows for the first individual. The same pattern was repeated for all individuals, each with 12 rows. The index variable preserved the order of the relative warps such that comparisons between groups (e.g., high predation/low predation) were made by matching each relative warp to the same relative warp in each group (i.e., relative warp 1 in the high predation environment was compared to relative warp 1 in the low predation environment). Our main goal was to determine how predation environment affects body shape; thus, it is the two‐way interaction of the predation environment and the index variable that tested the hypothesis of interest (i.e., does shape vary on at least some of the relative warps between predation environments). Main effects by themselves test only for an average effect across all relative warps. Because relative warps are principal components, they have a mean of 0; and more importantly, they have an arbitrary ordination. Thus, a single individual may have a positive score on some relative warps and a negative score on other relative warps so that their mean score across all relative warps may be near 0. It was only by matching relative warps in the same order (by using the index variable as a predictor) that we could accurately test the hypothesis of interest (Hassell et al., 2012; Ingley et al., 2014; Roth‐Monzón et al., 2020; Searle et al., 2021; Wesner et al., 2011). We estimated degrees of freedom using the Kenward and Roger method (1997). We used Proc MIXED in SAS to run this analysis (SAS version 9.4; SAS Institute Inc., Cary, NC, USA).

To visualize the effects of predation environment on shape, we calculated a divergence vector (Langerhans, 2009; Langerhans & Makowicz, 2009) that characterizes differences in shape across all relative warps for discrete predictor variables. We calculated this divergence vector by summing the products of the first eigenvector (from a principal components analysis of the least squares means for each relative warp in the two predation environments) multiplied by the associated relative warp scores for each fish. We then regressed divergence scores for each individual on their respective shape variables in tpsRegr (Rohlf, 2003b) to generate thin‐plate spline visualizations of the extremes of shape variation between predation environments. Resulting thin‐plate splines represent shape divergence across all relative warps between predation environments.

## 3 RESULTS

**FIGURE 3:**
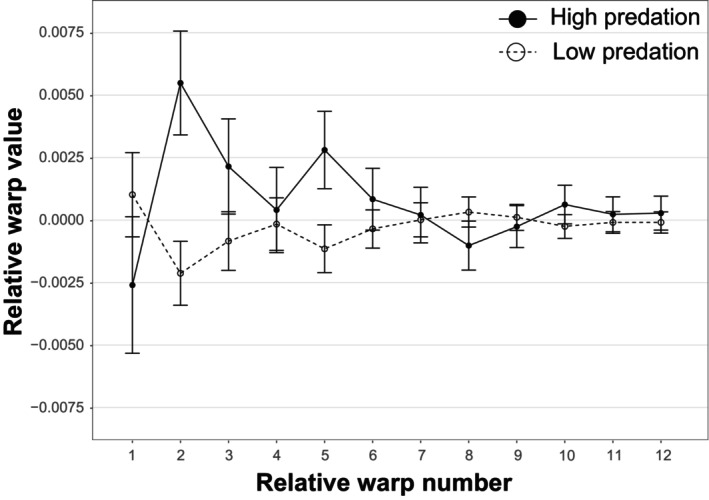
Least squares means of relative warps 1–12 for the lateral view of the body of *Alfaro cultratus* (error bars represent 95% confidence intervals of the mean). Low predation environment is represented by open circles and dashed lines, and high predation environment is represented by closed circles and solid lines.

**FIGURE 4:**
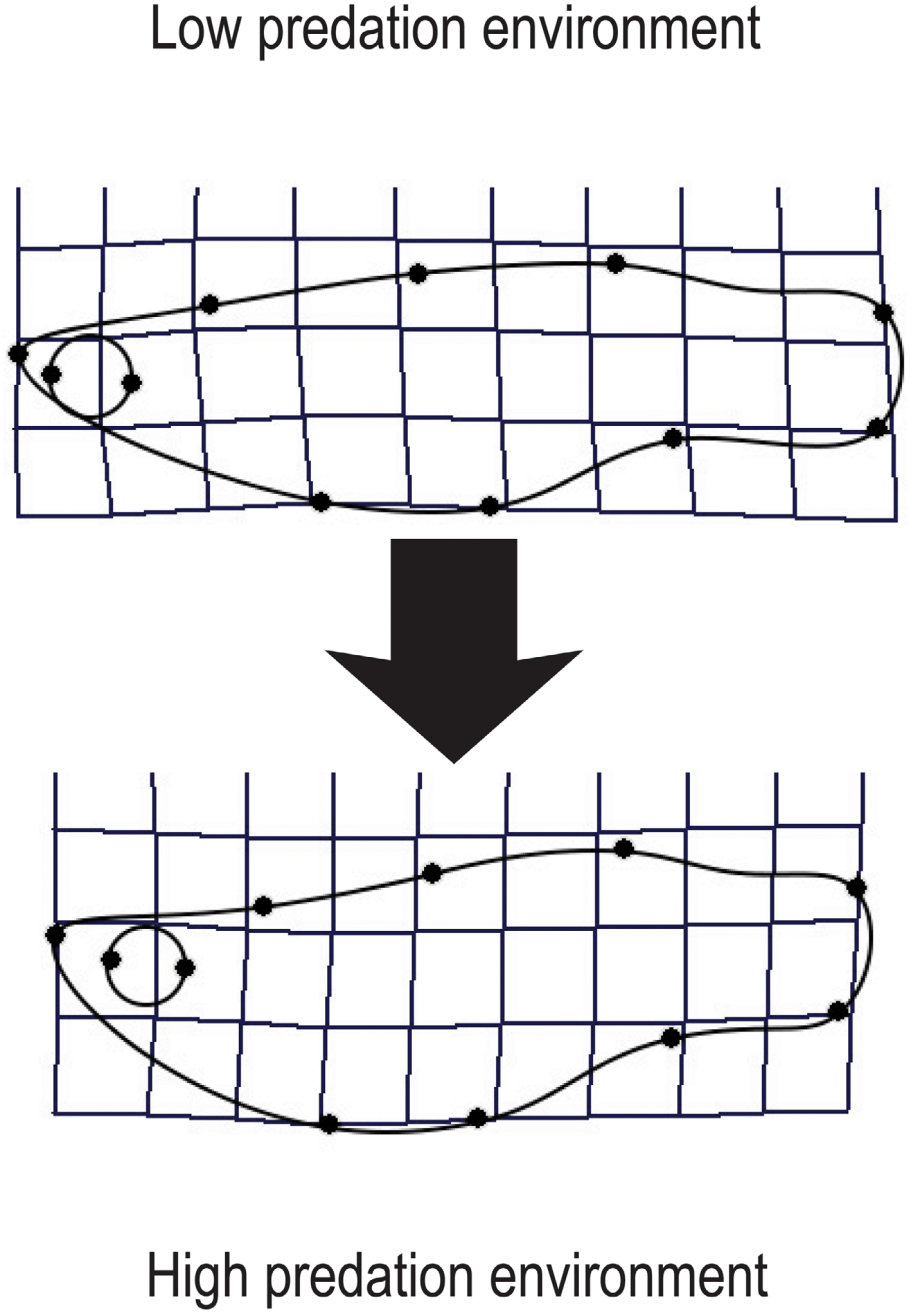
Thin‐plate splines representing the extremes of body shape variation in response to predation environment.

Predation environment had a significant effect on body shape as indicated by the significant interaction with the index variable (Table 2). The covariates stream gradient and centroid size each also accounted for significant variation in body shape (Table 2). Relative warps 1, 2, 3, 5, 8, and 10 showed significant differences between high predation and low predation environments (Figure 3). Fish in high predation environments exhibited deeper and shorter bodies and a deeper but shorter caudal peduncle area relative to those in low predation environments. In addition, fish in high predation environments had relatively larger heads and a longer rostrum, and the eye shifted more posterior and dorsal compared to fish in low predation environments (Figure 4).

**TABLE 2:** Multivariate analysis of covariance effects for body shape (type 3 table).

Body shape	Degrees of freedom, num/den	*F*‐value	*p*‐Value
Predation	1/46.5	14.69	.0004
Gradient	1/52.3	15.63	.0002
Centroid size (CS)	1/449	6.02	.0145
Index	11/2137	3.31	.0002
Predation*Index	11/2137	6.58	<.0001
Gradient*Index	11/2137	2.05	.0211
CS*Index	11/2137	3.12	.0004

## 4 DISCUSSION

Body shape in female *A. cultratus* differs significantly between high and low predation environments. However, the way in which shape differs between predation environments is not consistent with patterns found in other fish and specifically livebearing fish systems. Typically, livebearers from high predation environments exhibit a relatively more elongate body, longer and deeper caudal peduncle, shallower anterior head or body region, and a lower eye position than fish in the same species from low predation sites (Ingley et al., 2014; Langerhans, 2009; Langerhans & DeWitt, 2004; Langerhans & Makowicz, 2009). The deeper caudal peduncle is considered an adaptation for predator avoidance, and it has been shown in experimental studies to result in faster burst‐swimming speed (Langerhans et al., 2004). The slab‐sided body shape of *A. cultratus* appears to be a hydrodynamic adaptation for stabilized swimming because it reduces turbulence and thus energetic costs (Araújo et al., 2017; Belk et al., 2011; Golden et al., 2021) when swimming in high velocities. At sites where *A. cultratus* co‐occurs with predators, we also observed enlargement of the caudal peduncle but without the accompanying elongated body. Instead, our sample showed a shortening of the body along with deepening of the head and a more dorsal eye position, in the presence of predators. We hypothesize that this shape combination is due to the interaction between adaptations for steady swimming at high river currents and predator avoidance. This type of morphological shift in response to predators has not been well explored in other systems. Deepening of the body has been proposed as an adaptation to avoid predation from gape‐limited piscivores that eat prey whole and by increasing handling time that provides greater opportunity for escape (Belk & Hales Jr, 1993; Brönmark & Miner, 1992; Portz & Tyus, 2004; Williams et al., 2017). The increase in the anterior body depth of *A. cultratus* may function as an antipredator adaptation against the relatively small predators that inhabit the small streams where this species occurs. The distribution of body sizes of prey and gape sizes of predators would be a fruitful area for future research to determine if gape limitation is important in these systems.

Although the effect of predation on life history is a consequence of differential mortality among age or size classes (Johnson & Bagley, 2011), predation affects body shape by giving selective advantages to those individuals whose morphologies allow them to evade predation either by avoiding predation by gape size limited predators, improving burst speed, or having better predator detection. Among the populations included in this study, *A. cultratus* exhibits no differences in life history traits (Golden et al., 2021). This lack of difference in life history traits is strikingly different from patterns found in other poecilid species (Downhower et al., 2000; Golden et al., 2021; Jennions et al., 2006; Johnson & Bagley, 2011; Johnson & Belk, 2001; Reznick & Endler, 1982). Lack of response to predation in life history traits was hypothesized to be due to a shape constraint preventing the divergence because of the species having evolved a narrow body and ventral keel that might be selected for efficient swimming in the environments they live in regardless of predation (Golden et al., 2021). The morphometric results further support the constraint hypothesis by suggesting that predation does have an effect in the species, but that the constraint for efficient swimming might be causing shape in *A. cultratus* to differ in a nontypical way between predation environments. These results seem to indicate that life history is more restrained than morphology in narrow‐bodied species. Piscivores in this system might not be exerting preferential mortality on a specific size class (Johnson & Bagley, 2011), but they may be selectively consuming shallow‐bodied individuals that fit within their gape. This sort of selection by predators could lead to the patterns in body shape observed here. In addition, gape‐limited predation can result in differences in size among populations, and as noted above size can be related to life history variation among populations. However, in this study, the range of individual length for each sample is highly overlapping between predator and non‐predator locations (Table 1), and in a previous study, there was no difference in mean size at maturity between predator and non‐predator sites for both males and females (Golden et al., 2021), suggesting that differences in shape are not a consequence of differences in size among locations. Gape‐limited predation, as we have discussed it here, relates mainly to piscivorous fishes. In contrast, the presence and densities of birds or invertebrate predators and their effects on body shape of fishes might represent an important effect for future consideration that has not been well studied yet.

The selective pressures behind the ventral keel and unusual shape of female *A. cultratus* are still not fully understood. Whether such patterns hold in males also presents an interesting question. The suggestion that this shape contributes to better stabilized swimming needs to be experimentally tested, and differences in performance in both steady swimming and burst swimming between low and high predation environment populations need to be compared to determine whether the differences in body shape observed in this study provide any sort of antipredator advantage.

## AUTHOR CONTRIBUTIONS

**Diego A. Ardon:** Data curation (supporting); formal analysis (equal); visualization (lead); writing – original draft (lead); writing – review and editing (lead). **Kaitlyn B. Golden:** Data curation (lead); investigation (lead); writing – review and editing (equal). **Trevor J. Williams:** Investigation (supporting); writing – review and editing (equal). **Mark C. Belk:** Conceptualization (equal); formal analysis (equal); methodology (lead); supervision (equal); writing – review and editing (equal). **Jerald B. Johnson:** Conceptualization (equal); supervision (equal); writing – review and editing (equal).

## CONFLICT OF INTEREST STATEMENT

None declared.

## Data Availability

Data are available at the following repository: 10.5061/dryad.c59zw3rdz (Reviewer sharing link: https://datadryad.org/stash/share/OVsB4Gbmd2MrQH0YpifwXW0m3ZTX4ghCQmqMEhpsX4E).

## References

[ece310787-bib-0001] Aguirre, W. E. , & Bell, M. A. (2012). Twenty years of body shape evolution in a threespine stickleback population adapting to a lake environment. Biological Journal of the Linnean Society, 105(4), 817–831. 10.1111/j.1095-8312.2011.01825.x

[ece310787-bib-0002] Anderson, T. W. (2003). An introduction to multivariate statistical analysis. Wiley. https://books.google.com/books?id=Cmm9QgAACAAJ

[ece310787-bib-0003] Andersson, J. , Johansson, F. , & Soderlund, T. (2006). Interactions between predator‐ and diet‐induced phenotypic changes in body shape of crucian carp. Proceedings of the Royal Society B: Biological Sciences, 273(1585), 431–437. 10.1098/rspb.2005.3343 PMC1560211 16615209

[ece310787-bib-0004] Araújo, M. S. , Layman, C. A. , & Langerhans, R. B. (2017). Body streamlining is related to higher growth in Bahamian mosquitofish. Evolutionary Ecology Research, 18(4), 383–391.

[ece310787-bib-0005] Belk, M. C. , & Hales, L. S., Jr. (1993). Predation‐induced differences in growth and reproduction of bluegills (*Lepomis macrochirus*). Copeia, 1993, 1034–1044.

[ece310787-bib-0006] Belk, M. C. , Ingley, S. J. , & Johnson, J. B. (2020). Life history divergence in livebearing fishes in response to predation: Is there a microevolution to macroevolution barrier? Diversity‐Basel, 12(5), 179. 10.3390/d12050179

[ece310787-bib-0007] Belk, M. C. , Nance, E. E. , & Johnson, J. B. (2011). Life history of *Brachyrhaphis parismina*: Variation within and among populations. Copeia, 2011(3), 372–378.

[ece310787-bib-0008] Blake, R. W. (2004). Fish functional design and swimming performance. Journal of Fish Biology, 65(5), 1193–1222. 10.1111/j.0022-1112.2004.00568.x

[ece310787-bib-0009] Brönmark, C. , & Miner, J. G. (1992). Predator‐induced phenotypical change in body morphology in crucian carp. Science, 258(5086), 1348–1350. 10.1126/science.258.5086.1348 17778362

[ece310787-bib-0010] Burns, J. G. , Di Nardo, P. , & Rodd, F. H. (2009). The role of predation in variation in body shape in guppies *Poecilia reticulata*: A comparison of field and common garden phenotypes. Journal of Fish Biology, 75(6), 1144–1157. 10.1111/j.1095-8649.2009.02314.x 20738605

[ece310787-bib-0011] Bussing, W. A. (2002). Peces de las aguas continentales de costa rica = freshwater fishes of costa rica (2nd ed, Vol. 46). Editorial de la Universidad de Costa Rica.

[ece310787-bib-0012] Deacon, A. E. , Jones, F. A. M. , & Magurran, A. E. (2018). Gradients in predation risk in a tropical river system. Current Zoology, 64(2), 213–221. 10.1093/cz/zoy004 30402062 PMC5905555

[ece310787-bib-0013] Domenici, P. (2010). Fish locomotion: An eco‐ethological perspective. CRC Press.

[ece310787-bib-0014] Downhower, J. F. , Brown, L. P. , & Matsui, M. L. (2000). Life history variation in female *Gambusia hubbsi* . Environmental Biology of Fishes, 59, 415–428.

[ece310787-bib-0015] Golden, K. B. , Belk, M. C. , & Johnson, J. B. (2021). Predator environment does not predict life history in the morphologically constrained fish *Alfaro cultratus* (Cyprinodontiformes: Poeciliidae). Frontiers in Ecology and Evolution, 9, Article 607802. 10.3389/fevo.2021.607802

[ece310787-bib-0016] Haas, T. C. , Heins, D. C. , & Blum, M. J. (2015). Predictors of body shape among populations of a stream fish (*Cyprinella venusta*, Cypriniformes: Cyprinidae). Biological Journal of the Linnean Society, 115(4), 842–858. 10.1111/bij.12539

[ece310787-bib-0017] Hassell, E. M. A. , Meyers, P. J. , Billman, E. J. , Rasmussen, J. E. , & Belk, M. C. (2012). Ontogeny and sex alter the effect of predation on body shape in a livebearing fish: Sexual dimorphism, parallelism, and costs of reproduction. Ecology and Evolution, 2(7), 1738–1746. 10.1002/ece3.278 22957177 PMC3434940

[ece310787-bib-0018] Helfman, G. S. , Collette, B. B. , Facey, D. E. , & Bowen, B. W. (2009). The diversity of fishes: Biology, evolution, and ecology. John Wiley & Sons.

[ece310787-bib-0019] Ingley, S. J. , Billman, E. J. , Belk, M. C. , & Johnson, J. B. (2014). Morphological divergence driven by predation environment within and between species of *Brachyrhaphis* fishes. PLoS One, 9(2), Article e90274. 10.1371/journal.pone.0090274 24587309 PMC3936007

[ece310787-bib-0021] Jennions, M. D. , Wong, B. B. , Cowling, A. , & Donnelly, C. (2006). Life‐history phenotypes in a live‐bearing fish *Brachyrhaphis episcopi* living under different predator regimes: Seasonal effects? Environmental Biology of Fishes, 76, 211–219.

[ece310787-bib-0022] Johnson, J. B. (2002). Divergent life histories among populations of the fish *Brachyrhaphis rhabdophora*: Detecting putative agents of selection by candidate model analysis. Oikos, 96(1), 82–91. 10.1034/j.1600-0706.2002.960109.x

[ece310787-bib-0023] Johnson, J. B. , & Bagley, J. C. (2011). Ecological drivers of life‐history divergence. In J. P. Evans , A. Pilastro , & I. Schlupp (Eds.), Ecology and evolution of poeciliid fishes (pp. 38–49). The University of Chicago Press.

[ece310787-bib-0024] Johnson, J. B. , & Belk, M. C. (2001). Predation environment predicts divergent life‐history phenotypes among populations of the livebearing fish *Brachyrhaphis rhabdophora* . Oecologia, 126(1), 142–149. 10.1007/s004420000504 28547433

[ece310787-bib-0025] Johnson, J. B. , & Belk, M. C. (2020). Predators as agents of selection and diversification. Diversity‐Basel, 12(11), Article 415. 10.3390/d12110415

[ece310787-bib-0026] Johnson, J. B. , & Zúñiga‐Vega, J. J. (2009). Differential mortality drives life‐history evolution and population dynamics in the fish *Brachyrhaphis rhabdophora* . Ecology, 90(8), 2243–2252. 10.1890/07-1672.1 19739386

[ece310787-bib-0027] Jonsson, B. , & Jonsson, N. (2001). Polymorphism and speciation in Arctic charr. Journal of Fish Biology, 58(3), 605–638. 10.1111/j.1095-8649.2001.tb00518.x

[ece310787-bib-0028] Jourdan, J. , Krause, S. T. , Lazar, V. M. , Zimmer, C. , Sommer‐Trembo, C. , Arias‐Rodriguez, L. , Klaus, S. , Riesch, R. , & Plath, M. (2016). Shared and unique patterns of phenotypic diversification along a stream gradient in two congeneric species. Scientific Reports, 6(1), 38971. 10.1038/srep38971 27982114 PMC5159898

[ece310787-bib-0029] Kenward, M. G. , & Roger, J. H. (1997). Small sample inference for fixed effects from restricted maximum likelihood. Biometrics, 53(3), 983–997. 10.2307/2533558 9333350

[ece310787-bib-0030] Landy, J. A. , & Travis, J. (2015). Shape variation in the least killifish: Ecological associations of phenotypic variation and the effects of a common garden. Ecology and Evolution, 5(23), 5616–5631. 10.1002/ece3.1780 27069611 PMC4813119

[ece310787-bib-0031] Langerhans, R. B. (2008). Predictability of phenotypic differentiation across flow regimes in fishes. Integrative and Comparative Biology, 48(6), 750–768. 10.1093/icb/icn092 21669830

[ece310787-bib-0032] Langerhans, R. B. (2009). Trade‐off between steady and unsteady swimming underlies predator‐driven divergence in *Gambusia affinis* . Journal of Evolutionary Biology, 22(5), 1057–1075. 10.1111/j.1420-9101.2009.01716.x 21462405

[ece310787-bib-0033] Langerhans, R. B. , & DeWitt, T. J. (2004). Shared and unique features of evolutionary diversification. The American Naturalist, 164(3), 335–349. 10.1086/422857 15478089

[ece310787-bib-0034] Langerhans, R. B. , Layman, C. A. , Shokrollahi, A. M. , & DeWitt, T. J. (2004). Predator‐driven phenotypic diversification in *Gambusia affinis* . Evolution, 58(10), 2305–2318. 10.1111/j.0014-3820.2004.tb01605.x 15562692

[ece310787-bib-0035] Langerhans, R. B. , & Makowicz, A. M. (2009). Shared and unique features of morphological differentiation between predator regimes in *Gambusia caymanensis* . Journal of Evolutionary Biology, 22(11), 2231–2242. 10.1111/j.1420-9101.2009.01839.x 20069725

[ece310787-bib-0036] Lauder, G. V. , & Drucker, E. G. (2002). Forces, fishes, and fluids: Hydrodynamic mechanisms of aquatic locomotion. Physiology, 17(6), 235–240. 10.1152/nips.01398.2002 12433977

[ece310787-bib-0060] Mercer, M. , Searle, P. C. , Cifuentes, R. , Habit, E. & Belk, M. C. (2020). Morphometric response of galaxias maculatus (Jenyns) to Lake Colonization in Chile. Diversity, 12, 219. 10.3390/d12060219

[ece310787-bib-0037] Merigoux, S. , & Ponton, D. (1998). Body shape, diet and ontogenetic diet shifts in young fish of the Sinnamary River, French Guiana, South America. Journal of Fish Biology, 52(3), 556–569. 10.1006/jfbi.1997.0599

[ece310787-bib-0038] Meyer, A. (1990). Morphometrics and allometry in the trophically polymorphic cichlid fish, *Cichlasoma citrinellum*: Alternative adaptations and ontogenetic changes in shape. Journal of Zoology, 221(2), 237–260. 10.1111/j.1469-7998.1990.tb03994.x

[ece310787-bib-0039] Meyers, P. J. , & Belk, M. C. (2014). Shape variation in a benthic stream fish across flow regimes. Hydrobiologia, 738(1), 147–154. 10.1007/s10750-014-1926-1

[ece310787-bib-0040] Moccetti, P. , Rodger, J. R. , Bolland, J. D. , Kaiser‐Wilks, P. , Smith, R. , Nunn, A. D. , Adams, C. E. , Bright, J. A. , Honkanen, H. M. , & Lothian, A. J. (2023). Is shape in the eye of the beholder? Assessing landmarking error in geometric morphometric analyses on live fish. PeerJ, 11, e15545. 37605749 10.7717/peerj.15545 PMC10440062

[ece310787-bib-0041] Portz, D. , & Tyus, H. (2004). Fish humps in two Colorado River fishes: A morphological response to cyprinid predation? Environmental Biology of Fishes, 71(3), 233–245. 10.1007/s10641-004-0300-y

[ece310787-bib-0042] Reznick, D. , & Endler, J. A. (1982). The impact of predation on life history evolution in Trinidadian guppies (*Poecilia reticulata*). Evolution, 36(1), 160–177. 28581096 10.1111/j.1558-5646.1982.tb05021.x

[ece310787-bib-0043] Reznick, D. N. , & Travis, J. (2019). Experimental studies of evolution and eco‐evo dynamics in guppies (*Poecilia reticulata*). Annual Review of Ecology, Evolution, and Systematics, 50, 335–354.

[ece310787-bib-0044] Robinson, B. W. , & Wilson, D. S. (1994). Character release and displacement in fishes: A neglected literature. The American Naturalist, 144(4), 596–627.

[ece310787-bib-0045] Rohlf, F. (2003a). TpsDig, digitize landmarks and outlines, version 1.39. Department of Ecology and Evolution, State University of New York at Stony Brook.

[ece310787-bib-0046] Rohlf, F. (2003b). tpsRegr, shape regression. Department of Ecology and Evolution, State University of New York at Stony Brook.

[ece310787-bib-0047] Rohlf, F. (2003c). TPSRELW, version 1.29. Department of Ecology and Evolution, State University of new York at Stony Brook.

[ece310787-bib-0048] Rohlf, F. J. (2015). The tps series of software. Hystrix, 26(1), 9–12.

[ece310787-bib-0049] Rohlf, F. J. , & Slice, D. (1990). Extensions of the Procrustes method for the optimal superimposition of landmarks. Systematic Biology, 39(1), 40–59.

[ece310787-bib-0050] Roth‐Monzón, A. J. , Belk, M. C. , Zúñiga‐Vega, J. J. , & Johnson, J. B. (2020). Beyond pairwise interactions: Multispecies character displacement in Mexican freshwater fish communities. The American Naturalist, 195(6), 983–996. 10.1086/708513 32469659

[ece310787-bib-0051] Ruber, L. , & Adams, D. C. (2001). Evolutionary convergence of body shape and trophic morphology in cichlids from Lake Tanganyika. Journal of Evolutionary Biology, 14(2), 325–332. 10.1046/j.1420-9101.2001.00269.x

[ece310787-bib-0052] Schluter, D. (1993). Adaptive radiation in sticklebacks: Size, shape, and habitat use efficiency. Ecology, 74(3), 699–709. 10.2307/1940797

[ece310787-bib-0053] Schluter, D. , & McPhail, J. D. (1992). Ecological character displacement and speciation in sticklebacks. The American Naturalist, 140(1), 85–108. 10.1086/285404 19426066

[ece310787-bib-0054] Searle, P. C. , Mercer, M. , Habit, E. , & Belk, M. C. (2021). Ontogenetic shape trajectory of *Trichomycterus areolatus* varies in response to water velocity environment. PLoS One, 16(6), Article e0252780. 10.1371/journal.pone.0252780 34115773 PMC8195363

[ece310787-bib-0055] Smith, T. B. , & Skulason, S. (1996). Evolutionary significance of resource polymorphisms in fishes, amphibians, and birds. Annual Review of Ecology and Systematics, 27, 111–133.

[ece310787-bib-0056] Webb, P. W. (1984). Body form, locomotion and foraging in aquatic vertebrates. American Zoologist, 24(1), 107–120.

[ece310787-bib-0057] Wesner, J. S. , Billman, E. J. , Meier, A. , & Belk, M. C. (2011). Morphological convergence during pregnancy among predator and nonpredator populations of the livebearing fish *Brachyrhaphis rhabdophora* (Teleostei: Poeciliidae). Biological Journal of the Linnean Society, 104(2), 386–392. 10.1111/j.1095-8312.2011.01715.x

[ece310787-bib-0058] Williams, T. J. , Johnson, J. B. , & Belk, M. C. (2017). Interaction between predation environment and diet constrains body shape in Utah chub, *Gila atraria* (Cypriniformes: Cyprinidae). Biological Journal of the Linnean Society, 122(1), 147–156. 10.1093/biolinnean/blx050

[ece310787-bib-0059] Zelditch, M. , Swiderski, D. , & Sheets, H. D. (2012). Geometric morphometrics for biologists: A primer. Academic Press.

[ece310787-bib-0020] Zúñiga‐Vega, J. , Reznick, D. N. , & Johnson, J. B. (2007). Habitat predicts reproductive superfetation and body shape in the livebearing fish *Poeciliopsis turrubarensis* . Oikos, 116(6), 995–1005. 10.1111/j.0030-1299.2007.15763.x

